# A dermatologist guide to immunogenicity^☆^^☆☆^

**DOI:** 10.1016/j.ijwd.2016.05.001

**Published:** 2016-07-18

**Authors:** Collin M. Blattner, Soham P. Chaudhari, John Young, Jenny E. Murase

**Affiliations:** a Good Samaritan Regional Medical Center, Corvallis, Oregon; b Hackensack University Medical Center Palisades, North Bergen, New Jersey; c Department of Dermatology, Silver Falls Dermatology, Oregon; d Department of Dermatology, University of California, San Francisco, San Francisco, California; e Department of Dermatology, Palo Alto Foundation Medical Group, Mountain View, California

**Keywords:** antidrug antibodies, autoantibodies, biologic fatigue, HACA antibodies, resistance to biologics

## Abstract

Dermatologists should be aware that autoantibody formation may occur after the initiation of biologic therapy. This phenomenon has been referred to as immunogenicity and biologic fatigue. Because of this, patients may experience loss of clinical efficacy to a particular drug. To combat this phenomenon, low-dose immunomodulators may be used in hopes of preventing autoantibodies. We review the current literature and provide a basic treatment algorithm for patients with moderate to severe psoriasis.

## Introduction

Biologic agents were marketed to dermatologists as single drug therapy, but when marketed to gastroenterologists and rheumatologists, they were recommended as a supplement to other medications. Manufacturers eventually recognized that immunomodulators like methotrexate (MTX), azathioprine (AZA), and mercaptopurine (6-MP) reduced the production of autoantibodies against tumor necrosis factor alpha (TNFα) agents (Remicade, 2013). These data suggested that supplemental therapy with immunomodulators might prolong the efficacy of biologics when compared to TNFα monotherapy.

Biologics (with the exception of infliximab) are fully human monoclonal antibodies, so the immune response theoretically should be quiescent (Scott and De Groot, 2010). However, the immune system can unexpectedly produce anti-drug antibodies (ADA), resulting in decreasing efficacy of the biologics (Scott and De Groot, 2010). This effect was found among many biologics (the anti-TNFα agents adalimumab, infliximab and etanercept, and the anti-p40 [interleukin 12/23] ustekinumab), and many patients have been placed on an alternate biologic treatment regimen. The loss of efficacy has been partially attributed to neutralizing ADA formation against the biologic drug. These ADA lead to the formation of drug-antibody complexes that accelerate drug clearance from the circulation and subsequently inhibit function (Carrascosa et al., 2014).

Several factors may contribute to immunogenicity, including some that are extrinsic to the molecule (Scott and De Groot, 2010). Intravenous administration favors immunogenic tolerance, while subcutaneous or intramuscular injection favors a secondary immune response to inflammation from the injection site and drainage to local lymph nodes (Scott and De Groot, 2010). Antigen presenting cells (dendritic cells) are abundant in the skin, but the patient’s human leukocyte antigen type governs whether T-cell epitopes derived from the biologic are presented to T cells (Scott and De Groot, 2010). Patients with psoriasis (PsO) may have an inherent T-cell defect that increases immunity to a given drug; that is, the disease promotes immunogenic responses to proteins that might not normally trigger one (Scott and De Groot, 2010). A brief discussion regarding the use of combining biologics and MTX for the treatment of Crohn’s disease and rheumatoid arthritis (RA) are also provided.

## Methods

A systematic English-language literature search was conducted of both PubMed and MEDLINE databases from inception through December 10, 2014, to identify trials of biologics and MTX for the treatment of PsO and psoriatic arthritis (PsA). Key search terms included “biologic fatigue in psoriasis” and “immunogenicity in psoriasis”. Search terms were also used in combinations. The initial search yielded 76 articles. Articles that did not mention treatment of the disease, were not in English, and were not available online were excluded. After critical evaluation of the remaining articles by two investigators (C.B. and S.C.), we selected 18 articles to be included in this manuscript.

## Results

Concomitant use of MTX with a biologic reduces immunogenicity. Therefore, when MTX or other immunosuppressant therapies are administered in combination with biologic agents, the biologic has more durable efficacy. Key findings from selected PsO trial are summarized in Table 1.

### Psoriasis and psoriatic arthritis

PsO is a chronic, inflammatory, T-cell-mediated autoimmune disease that results from activation of inflammatory cytokines (interferon gamma, TNFα, IL-12, IL-23) and presents with erythematous scaly plaques in 1% to 3% of Caucasians (Saraceno et al., 2009). Almost 40% of psoriatic patients go on to develop PsA (Saraceno et al., 2009).

MTX is the most commonly used systemic treatment for moderate-to-severe plaque PsO in Europe (Boffa, 2005, Mikuls and O’Dell, 2000). It is thought to act as an immunosuppressant that targets lymphoid cell functions (Jeffes et al., 1995, Naldi and Griffiths, 2005, Schön and Boehncke, 2005) and reduces disease severity by at least 50% (Boffa, 2005, Mikuls and O’Dell, 2000). However, side effects include hematological malignancy, nonalcoholic fatty liver disease, and lung disease. Careful monitoring of hepatic, renal, and hematological labs is necessary, along with a liver biopsy if the total cumulative dose reaches 1.5 g.

Despite MTX therapy, some patients may experience little to no decrease in disease severity, warranting use of biologic therapies. While biologics have revolutionized treatment for resistant PsO, monotherapy with TNFα inhibitors may only be extremely effective for a period of time. PsO patients may experience a diminished clinical response as time progresses despite the same dosing schedule (biologic fatigue). This subsequent decrease in effectiveness is due to the development of ADA that may be prevented or reduced with combination therapy.

Studies have demonstrated that combining adalimumab with MTX can reduce the formation of ADA (Bartelds et al., 2007; Pathirana et al., 2009). Philipp et al. (2012) retrospectively studied patients with psoriatic skin disease. All were given adalimumbab with another systemic antipsoriatic drug and received on average 3.9 antipsoriatic therapies (phototherapy or systemic medications) before beginning combination therapy with adalimumab and a traditional systemic agent (Philipp et al., 2012). Combination therapy with MTX was most common (n = 32), followed by acitretin (n = 4) and cyclosporine (CsA) (n = 3). Combination therapy was effective in the majority of patients: 30/39 (76.9 %) had an excellent (n = 21) or good response (n = 9) (Philipp et al., 2012). This study further substantiated the belief that the early combination of MTX with adalimumab, and likely other biologics, could prevent immunogenicity in some patients (Philipp et al., 2012). Their retrospective analysis revealed that combination therapy with adalimumab and MTX prevented anti-adalimumab antibodies in 3/32 patients (Philipp et al., 2012). The combination of MTX and adalimumab was effective in 17/20 patients; treatment was either started with adalimumab and MTX simultaneously or adalimumab was added after MTX therapy had already begun. In patients who did not respond adequately to adalimumab alone, the addition of MTX led to a good or very good response (8/12 patients). The study also revealed a good safety profile, with no adverse events (AEs) severe enough to require hospitalization (Philipp et al., 2012). The most commonly reported side effects were upper respiratory tract infection (n = 5), bronchitis (n = 2), and influenza (n = 1) (Philipp et al., 2012).

CsA is another immunosuppressant that induces double strands breaks in DNA and may be associated with an increased risk of malignancies (Patel et al., 2009). Consequently, combination CsA and adalimumab therapy should be reserved for temporary rescue therapy or for severe recalcitrant pustular PsO. Although no large studies on combination therapy with CsA and adalimumab in PsO patients are available, case reports, case series, and open studies on patients with PsO/PsA have been documented with etanercept (D'Angelo et al., 2010, Kress, 2006, Lee et al., 2010, Yamauchi and Lowe, 2006). In these reports, combination therapy consisting of etanercept and CsA was effective and did not lead to serious AEs (D'Angelo et al., 2010, Kress, 2006, Lee et al., 2010, Yamauchi and Lowe, 2006). Further studies on the synergistic effects of CsA and TNF antagonists are needed.

Acitretin is a second-generation, systemic retinoid with no immunosuppressive effects (Lee and Koo, 2005). Acitretin is safe for long-term use and has no time limit restrictions, which makes it useful for combination and maintenance therapy (Lee and Koo, 2005). Three out of four patients who received combination therapy with adalimumab and acitretin in case series by Philipp et al. (2012) had a favorable response. Two previous reports of combination therapy with ADA and acitretin also demonstrated tolerability and efficacy (Conley et al., 2006, Smith et al., 2008). In addition, a clinical study of etanercept 25 mg/weekly combined with acitretin 0.4 mg/kg of body weight daily found this regimen to be just as effective as etanercept 25 mg twice weekly and more effective than acitretin monotherapy (Gisondi et al., 2008). All of these findings suggest that a combination of adalimumab and traditional systemic anti-PsO treatments, especially MTX and acitretin, are promising methods for managing severe or recalcitrant PsO.

In another study by Dalaker and Bonesrønning (2009), the effectiveness of infliximab in combination with MTX or AZA was evaluated for moderate-to-severe PsO. All patients had previously failed treatment with MTX and ultraviolet B phototherapy. Thirteen of 23 patients had tried biologic agents other than infliximab (12 etanercept, 7 efalizumab, 5 both etanercept and efalizumab, and 1 adalimumab). A 3-week washout period was done for all patients receiving another biological agent before infliximab was administered. Interestingly, one patient, a 6-year-old boy, safely received infliximab 5 mg/kg instead of 3 mg/kg in combination with MTX as advised by his pediatrician to reduce the risk of developing antibodies against infliximab during therapy (Dalaker and Bonesrønning, 2009).

Five patients who did not tolerate MTX were given AZA 50 mg/day in combination with infliximab 5 mg/kg, a regimen used for treating Crohn’s disease. Treatment with AZA and infliximab was initiated simultaneously.

Patient data was available for a minimum of 4 weeks and up to 5 years and 5 months. Only two patients stopped infliximab therapy secondary to loss of response after 14 months and 3 years, respectively. Both were receiving infliximab with MTX. At week 14, 91.3% of patients achieved psoriasis area score index (PASI) 50, 69.6% achieved PASI 75, and 39.1% achieved PASI 90 (Dalaker and Bonesrønning, 2009). There was a slight drop in efficacy after 1 year, but the overall effect of combination therapy was better than in studies with standard monotherapy (Menter et al., 2007, Reich et al., 2005). During maintenance therapy, the approach to loss of effectiveness was to shorten treatment intervals and/or increase the dose of MTX. The authors aimed to use the lowest effective dose of both MTX and infliximab, as therapy is generally long term. This approach maintained efficacy and was well tolerated, supporting the supplementation of biologics with low dose MTX (Eklund et al., 2003).

Infliximab is a chimeric antibody that can promote formation of neutralizing antibodies that may interfere with safety and efficacy of the drug. Formation of antibodies is associated with infusion reactions, lower postinfusion infliximab serum levels, and a shortened duration of response (Maini et al., 1998, Rutgeerts et al., 2006). Concomitant use of immunosuppressants (MTX, AZA) reduces the formation of antibodies against infliximab and improves the pharmacokinetics of the drug, to enhance clinical efficacy. Although MTX and AZA may be equally effective in enhancing efficacy, additional studies must be performed to draw further conclusions (Rutgeerts et al., 2006).

ADA were observed in 20 to 36% of psoriatic patients treated with infliximab 5 mg/kg every 8 weeks for 1 year in a large multicenter trial (Remicade, 2013). In another study by Adişen et al. (2010), mechanisms responsible for loss of clinical efficacy to infliximab were studied. The murine binding portion that comprises 25% of the antibody is antigenic in infliximab and is believed to cause immunogenicity to the drug (Baert et al., 2007, Cheifetz and Mayer, 2005). To determine causation, peripheral blood samples were collected from patients prior to the study and then before each infliximab infusion. Antibodies to infliximab (ATI) were found in 5 (33.3%) of 15 patients (Adişen et al., 2010). Each patient developed ATI at a different time (the fifth, sixth, seventh, 10th, and 13th infusions, respectively) after receiving an initial induction region of infliximab at week 0, 2, and 6, infliximab, and then every 8 weeks. In ATI positive patients, more infusions were necessary to reduce the mean PASI (5.9 ± 3.2 infliximab infusions achieved a decrease in the PASI score from a mean of 20.4 ± 8.3 to 5.3 ± 2.4 in ATI-negative patients versus 23.3 ± 11 to 10 ± 4.9 after 9 ± 5.2 infusions in ATI-positive patients) (Adişen et al., 2010). This finding showed that a similar numbers of infliximab infusions (*p* = .16) failed to satisfactorily reduce the mean PASI scores of ATI-positive patients (*p* = .1). Thus, the prevention of ATI is associated with a better clinical outcome. PsO patients may increase the efficacy of infliximab by adding MTX to their regimen, although more studies must be performed before definitive conclusions can be drawn.

Regularly scheduled maintenance infusions after an induction regimen or extending intervals between infusions may prolong the efficacy of infliximab by generating immunological tolerance (Maini et al., 1998, Nast et al., 2007, Rutgeerts et al., 2004, Sandborn, 2003). In concordance with this belief, Menter et al. (2007) found increased ATI formation in as-needed regimens (41.5%) compared to regularly administering infliximab every 8 weeks (35.8%). Moreover, induction regimen and maintenance therapy with 8-week intervals have been found better than the addition of an immunomodulator for preventing antibody formation (Cheifetz and Mayer, 2005). Despite the promising results of infliximab therapy, a patient’s medical history may preclude the provider from prescribing this agent.

In another study of severe PsO in which etanercept monotherapy was insufficient, Driessen et al. (2008) studied the effect of combining etanercept with conventional PsO treatments. Data were extracted from an existing database, and a case was defined as a patient using etanercept and MTX simultaneously for an indefinite period during follow-up. Introducing MTX after etanercept resulted in an improvement of PsO in four of six patients (66.7%). Eight patients were on MTX therapy before the start of etanercept, and five of six (83.3%) patients who discontinued MTX experienced a decrease in PASI. The results further established the benefits of combining etanercept and MTX when etanercept monotherapy fails to produce an adequate clinical outcome.

Infliximab accounts for the majority of cases of hepatitis B virus (HBV) reactivation and fulminant hepatitis, but rare cases of HBV reactivation with etanercept have been reported (Vilarrasa and Puig, 2014). Infliximab is a monoclonal antibody that neutralizes soluble and membrane bound TNFα, while etanercept is a fusion protein that only binds to soluble TNFα (Vilarrasa and Puig, 2014). Therefore, it may be beneficial to use etanercept instead of infliximab when a patient has a history of HBV. The use of etanercept has also been shown to enable tapering of MTX dose without increasing liver toxicity, infections, or myelosuppression in PsO patients (Yamauchi et al., 2005).

Zachariae et al. (2008) evaluated combination therapy for plaque PsO patients who previously failed MTX therapy. Patients with PASI ≥ 8 and/or total body surface are > 10% despite more than 3 months of MTX were randomized to either etanercept with MTX tapered and discontinued (n = 28) or etanercept with continuous MTX (n = 31). Patients received an average MTX dose of 13.7 mg per week. Significantly more patients had a Physicians' Global Assessment of "clear"/"almost clear" in the combination group compared with etanercept/MTX taper (66.7 vs. 37.0%; *p* = .025). Limiting factors for the study included a short duration of 24 weeks. The most common adverse side effect was infection, which was consistent with the findings of similar studies.

In another retrospective study by Antoniou et al. (2010), the sequential treatment of switching high-need PsO patients from efalizumab to etanercept was studied in 35 patients over a 4.5-year period. They evaluated the effectiveness and safety of etanercept as a sequential treatment in patients previously treated with efalizumab and different transition strategies from efalizumab to etanercept. After 24 weeks of etanercept therapy, 57% of patients had a PASI reduction of 75%, suggesting that alternating between biological agents is feasible. Three different approaches were utilized: etanercept in combination with CsA as bridge therapy, etanercept in combination with MTX as bridge therapy, or etanercept monotherapy (Antoniou et al., 2010). Combination therapy was efficacious in all patients, including eight patients with rebound phenomenon from efalizumab (Aksu et al., 2011, Antoniou et al., 2010, Chainani-Wu et al., 2014). From the study, it seemed that monotherapy with etanercept was not sufficient when transitioning from efalizumab in high-need patients with severe worsening or rebound PsO (Antoniou et al., 2010). In such patients, the combination of etanercept with CsA or MTX was more effective. Although efalizumab is no longer in use due to an increased risk of developing human John Cunningham polyomavirus (JC polyoma induced leukoencephalopathy), this study illustrates important considerations one must take when switching between different classes of biologics (Antoniou et al., 2010, Bellizzi et al., 2013).

Switching between different classes of biologics, including TNF ?thyc=5?> inhibitors and ustekinumab, is increasingly used, raising efficacy and safety questions. The first head-to-head study of biologics compared high-dose etanercept or ustekinumab (45 mg) in 903 patients with PsO. At week 12, 67.5% of ustekinumab-treated patients achieved a PASI 75, compared with 56.8% of etanercept-treated patients (Griffiths et al., 2010). Moreover, trials studying the sequential treatment from one biological agent to another may contribute to our understanding for managing complicated cases.

In another study by Gottlieb et al. (2012), patients with moderate to severe plaque PsO who had not failed prior MTX or TNF-inhibitor therapy were evaluated.^43^ Patients received etanercept plus MTX or etanercept monotherapy (etanercept 50 mg twice weekly for 12 weeks followed by 50 mg once weekly for 12 weeks). Patients were randomized 1:1 to receive MTX (7.5-15 mg weekly) or placebo. The primary endpoint was the proportion of patients achieving ≥ 75% improvement in PASI 75 at week 24.

PASI 75 was significantly higher at week 24 for the combination therapy group compared with the monotherapy group (77.3% vs. 60.3%; *p* < .0001) (Gottlieb et al., 2012). Other PASI improvement scores at week 12 (PASI 75, 70.2% vs. 54.3% [*p* = .01]; PASI 50, 92.4% vs. 83.8% [*p* = .01]; and PASI 90, 34.0% vs. 23.1% [*p* = .03]) showed similar results as did week 24 PASI 50 (91.6% vs. 84.6%; *p* = .01) and PASI 90 (53.8% vs. 34.2%; *p* = .01). Significantly more patients receiving combination therapy than monotherapy had static Physicians' Global Assessment of clear/almost clear at week 12 (65.5% vs. 47.0%; *p* = .01) and week 24 (71.8% vs. 54.3%; *p* = .01) (Gottlieb et al., 2012). AEs were reported in 74.9% and 59.8% of combination therapy and monotherapy groups, respectively; three serious AEs were reported in each arm. This demonstrated that combination therapy with etanercept plus MTX had acceptable tolerability and increased efficacy compared with etanercept monotherapy in patients with moderate to severe PsO.

In PsA studies, patients who were ATI positive had higher rates of drug clearance, reduced efficacy, and infusion reaction (Remicade, 2013). In another PsA study, 37% of 128 patients treated with anti-TNFα drug developed autoantibodies (Bardazzi et al., 2014). Almost half (48.48 %) of those who received infliximab developed autoantibodies (Bardazzi et al., 2014). Forty-five patients were switched to one or more additional TNF-α inhibitors, and 25 developed autoantibodies (Bardazzi et al., 2014).

### Crohn’s disease

In a systematic review of Crohn’s patients who received infliximab and immunomodulators (6-MP, AZA, MTX), a lower incidence of ADA was found in the combination therapy cohort (10% and 18%, respectively; *p* = .02) (Cassinotti and Travis, 2009). ATI were more common following drug free intervals greater than 16 weeks (Cassinotti and Travis, 2009). ATI were also associated with a 12% absolute increase in infusion reactions, but there was no increase in serious infusion reactions or serum sickness-like reactions (Cassinotti and Travis, 2009). Across all studies, those receiving concomitant immunodulators (6-MP, AZA, MTX) had a significantly lower incidence of infusion reactions (3%, 38/1174) compared with patients not receiving concomitant immunosuppressive therapy (6%, 171/2666; *p* < .001) (Cassinotti and Travis, 2009). These findings led the authors to conclude that reduced antibody formation was of clinical benefit.

### Rheumatoid arthritis

The use of TNF antagonists in combination with MTX is approved for multiple rheumatologic disorders (Humira, 2004). The largest prospective studies supporting the effects of combining MTX with biologic therapy have been done for RA. Patients in Studies RA-I, RA-II, and RA-III were tested at multiple time points for antibodies to adalimumab (Humira, 2004). Approximately 5% (58/1062) of patients developed low-titer antibodies to adalimumab at least once during the first year of treatment. Patients treated with concomitant MTX had a lower rate of antibody formation than those on adalimumab monotherapy (1% vs. 12%) (Humira, 2004). With monotherapy, patients receiving injections every other week were more likely to develop autoantibodies than those receiving weekly dosing (Humira, 2004).

In another study, anti-adalimumab antibodies were detected in 21/121 patients (17%) during 28 weeks of treatment (Bartelds et al., 2007). The European League Against Rheumatism stated that nonresponders had antibodies significantly more often than good responders (34% vs. 5%; *p* = .032) (Bartelds et al., 2007). Patients with antibodies showed less improvement in disease activity (mean [SD] ΔDAS28, 0.65 [1.35]) than patients without antibodies (mean [SD], ΔDAS28 1.70 [1.35]) (*p* = .001) (Bartelds et al., 2007). Patients with antibodies during follow-up also had lower serum adalimumab concentrations at 28 weeks than patients without antibodies (median 1.2 mg/l, range 0.0-5.6 vs. median 11.0 mg/l, range 2.0-33.0, respectively; *p* = .001) (Bartelds et al., 2007). Good responders had higher serum adalimumab concentrations than moderate responders (*p* = .021) and nonresponders (*p* = .001) (Bartelds et al., 2007). Concomitant MTX use was lower in the group with anti-adalimumab antibodies (52%) than in the group without antibodies (84%) (*p* = .003) (Bartelds et al., 2007).

With etanercept, antibodies to the TNFα receptor portion or other protein components of the drug were detected at least once in the sera of approximately 6% of adult patients with RA, PsA, ankylosing spondylitis, or plaque PoS (Enbrel, 2013). All antibodies were non-neutralizing, and as expected, the percentage of patients testing positive for autoantibodies increased as the duration of study was extended (Enbrel, 2013). However, no data were collected beyond 120 weeks (Enbrel, 2013).

The standard therapeutic regimen of infliximab for RA is 3 mg/kg in combination with MTX (Dalaker and Bonesrønning, 2009). Immunosuppression and episodic treatment ATI were studied in the ACCENT I trial (Hanauer et al., 2002). In patients receiving infliximab episodically, the rate of ATI development without immunosuppression was 38% compared with 16% in patients receiving immunomodulators (MTX, AZA). This difference was statistically significant (*p* < .001), but with maintenance dosing, the rate of ATI development was lower, and the effect of immunomodulators was less pronounced (Hanauer et al., 2002). In the 5 mg/kg every 8 week group, 7% of patients receiving immunomodulators developed ATI compared with 11% of patients not receiving immunosuppressants and in the 10 mg/kg 8-week group, 4% compared with 8%, respectively. However, the difference was not statistically significant between the maintenance groups and those receiving immunomodulators.

## Reasons for combination therapy with biologics

Reasons for starting combination therapy include lack of efficacy to traditional treatments, disease severity, suppressing antibody formation, or briefly overlapping treatment methods when switching therapies. When combination therapy is used from the beginning, the decision should be made based on severity of disease. Table 2 presents the half-life for traditional antipsoriatic drugs, and Table 3 reviews the same for each biologic agent discussed in this review. Table 4 provides the route of administration and dosing for each of these biologics.

Biologic treatments are largely devoid of liver toxicity. TNFα antagonists treatments have proven to be effective and safe in patients with chronic hepatitis C virus infections and other noninfectious chronic liver disorders, including alcoholic and nonalcoholic liver diseases. However, in chronic HBV, anti-TNFα treatments carry a high risk of HBV reactivation, especially infliximab. Anti-IL-12/23 treatments are also effective in patients with PsO, but data regarding their safety in chronic hepatitis infections are still limited.

## Conclusion

In summary, combination therapy with biologic and traditional systemic antipsoriatic drugs or ultraviolet light therapy if MTX fails to work is a treatment option for certain subgroups of PsO patients. This includes patients who do not respond adequately to traditional Ps0 therapies, those with severe PsO, and those with pustular or palmoplantar PsO. Combination therapy with MTX should be used to reduce the risk of developing ADA antibodies.

**Fig. 1:**
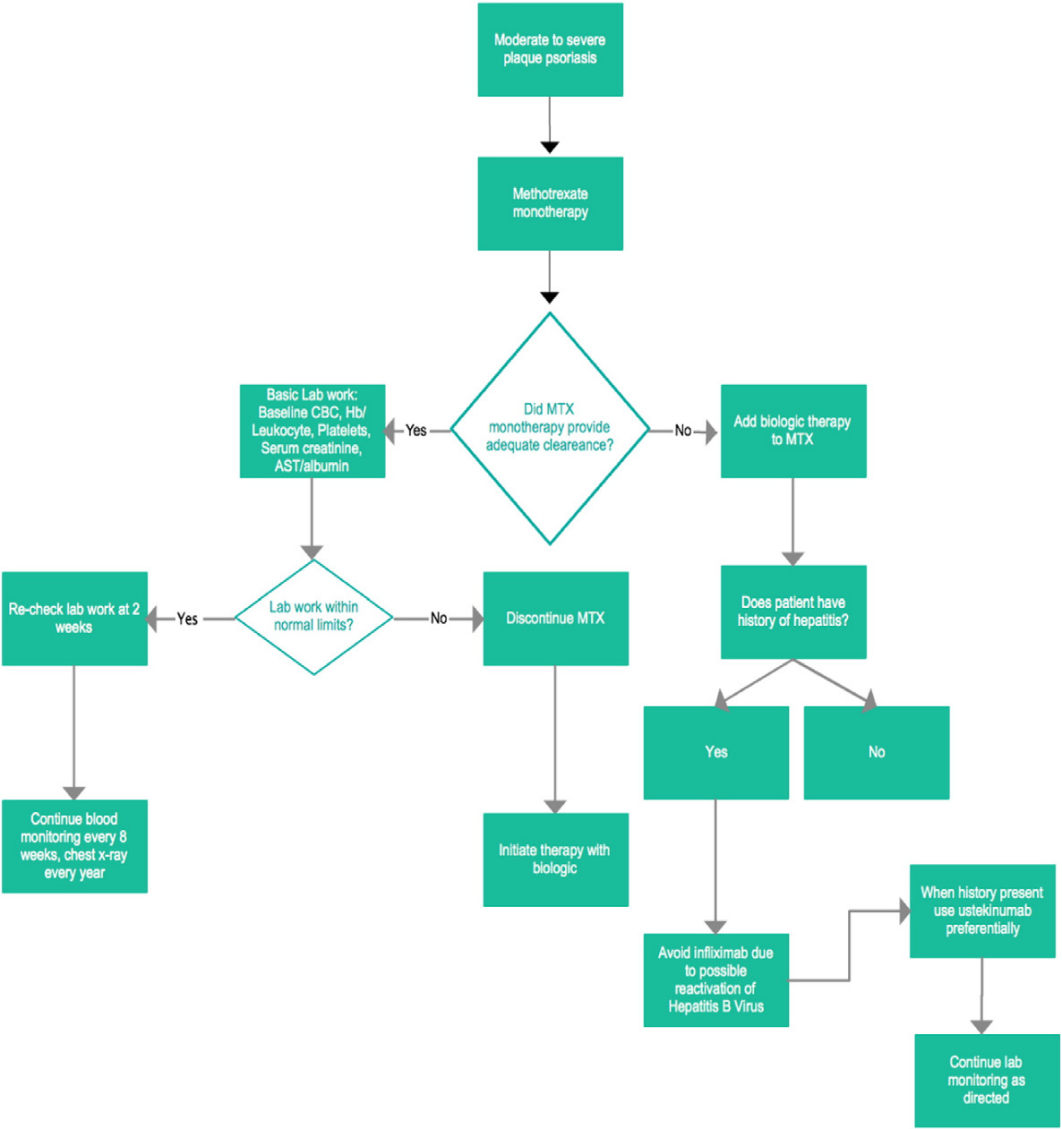
Basic treatment algorithm for moderate to severe psoriasis.

A basic treatment algorithm for patients with moderate PsO should include the initiation of MTX for at least 12 weeks to demonstrate response (Fig. 1). If MTX monotherapy is inadequate, then the addition of a biologic can be considered. Dermatologists should dose biologics without interruption and at intervals that make sense with regard to drug half-life. Concomitant use of MTX reduces immunogenicity. When given with MTX, biologic agents invariably show increased and more durable efficacy even when MTX is ineffective as monotherapy. A sensible practice is to add a biologic therapy to MTX, not vice versa, as immunogenicity may be difficult to reverse once it has occurred.

## Figures and Tables

**Table 1:** Selected publications on combination therapy of biologics with MTX in PsO

Reference	Study type	Number of patients with biologic and MTX/mean duratiof tx weeks	Biologic and average MTX dose/other systemic agent	Timing of MTX	Efficacy	Antibody Levels	Tolerability
Lopez-Ferrer et al. BrJ Dermatol 2013; 169; 1141-7	Retrospective	26/24	Adalimumab 40mg eow 5 ± 12.5mg MTX/week^2^	Add-on MTX when insufficient response to Adalimumab	After 24 weeks Combination group 73.5% PASI 75 67.5% PASI 90 Monotherapy group 43.5% PASI 75; 34.8% PASI 90	Did not measure	Infections, including de novo infection by Mycobacterium tuberculosis, accounted for most SAEs, and paradoxical flares of psoriasis and psoriatic arthritis were relatively frequent in daily clinical practice.
Philipp et al. J Dtsch Dermatol Ges 2012 10: 821-37	Retrospective	32/43	Adalimumab 40mg eow 12.4 ± 4.5mg MTX/week	20 patients received MTX concomitantly	85% PASI 50-75^1^	Prevention of anti-ADA antibodies was the reason in 3/32 patients for combination therapy with ADA and MTX.	Eighteen patients experienced 24 adverse events; none was severe and/or required hospitalization. More data are needed to determine the long-term safety and efficacy of these combinations
				12 patients received add on MTX when insufficient response to Adalimumab	67% PASI 50-75^1^		
Van den Reek et al. J Dermatolog Treat 2013; 24: 361-8	Prospective	11/24	Adalimumab 40mg eow 9.5 ± 3.2mg MTX/week	Add-on MTX when insufficient response to adalimumab	After 12 weeks 9% PASI 50 After 24 weeks 18% PASI 50	Twenty-five percent of first treatment episodes with adalimumab dose escalation induced a PASI50 response after 12 weeks and 35% after 24 weeks. Addition of MTX to adalimumab every other week resulted in PASI50 in 9% after 12 weeks and 18% after 24 weeks.	No related serious adverse events were reported.
Dalaker et al. J Eur Acad Dermatol Venereol 2009; 23: 277-82	Retrospective	18/106	Infliximab 3-5mg/kg 11.66 mg MTX/week^2^	MTX started concomitantly	After 14 weeks 91.3% PASI 50 69.6% PASI 75 39.1% PASI 90 After 1 year 80% PASI 50 60% PASI 75 33.3% PASI 90	6-year-old boy, received infliximab 5 mg/kg instead of 3 mg/kg in combination with methotrexate EXPRESS trial with 5 mg/kg infliximab monotherapy with detectable preinfusion serum infliximab concentrations: maintained maintained their PASI 75 response over time; \ undetectable serum infliximab concentrations (below 0.1 g/mL), less likely to maintain response. 5	Combination regimens of infliximab with methotrexate or azathioprine were well tolerated, and only one patient discontinued therapy because of an adverse event (lung embolism) after two infusions with infliximab.
Driessen et al. Br J Dermatol 2008; 159: 460-3	Prospective	14/40	Etanercept 50mg twice weekly the first 12 weeks, than 25mg twice tweekly 12.5mg^2^ MTX/week	8 patients started with MTX and received add-on etanercept	Discontinuation of MTX in 6 of these patients resulted in a decrease in clinical efficacy in 5 patients^3^	Did not measure	Etanercept combined with methotrexate was well tolerated, and only mild adverse events were reported.
				6 patients recived add-on MTX when insufficient response to etanercept	67% improvement efficacy^3^		
Zachariae et al. Acta Derm Venereol 2008; 88: 495-501	Prospective	31/24	Etanercept 50mg twice weekly the first 12 weeks, than 25mg twice weekly 13.4mg^2^ MTX/week	Add-on etanercept when insufficient response to MTX	After 24 weeks combination group 76.4% PASI 75	Did not measure	Very little difference between the two groups; The most common organ system class affected by adverse events was infections, where 7 (25.0%) and 12 (38.7%) adverse events were reported for the etanercept/methotrexate taper and combination groups, respectively.
				Etanercept with MTX tapered treatment	Tapered MTX group 51.3% PASI 75		
Antoniou et al. J Eur Acad Dermatol Venereol 2010; 24: 1413-20	Retrospective	11/24	Etanercept 50mg twice weekly the first 12 weeks, then 25 mg twice weekly 15mg MTX/week^2^	MTX started concomitantly	After 24 weeks Combination group 36.4% PASI 75 27.2% PASI 50 Monotherapy group 41.7% PASI 75 8.3% PASI 50	Did not measure	Etanercept was generally well tolerated. Laboratory abnormalities included hyperlipidaemia. Discontinued in two patients as a result of serious adverse events that consisted of an oral squamous cell carcinoma and a diffuse B-cell-non-Hodgkin lymphoma.
Gottlieb et al. Br J Dermatol 2012; 167: 649-57	Prospective	239/24	Etanercept 50mg twice weekly the first 12 weeks, then 25 mg twice weekly MTX ranging from 7.5-15mg/week	MTX started concomitantly	After 12 weeks Combination group 70.2% PASI 75 Monotherapy group 54.3% PASI 75 After 24 weeks combination group 77.3% PASI 75 Monotherapy group 60.3% PASI 75	Did not measure	More patients in the combination arm than in the monotherapy arm experienced at least 1 AE (74Æ9% vs. 59Æ8%), but most AEs were mild or moderate in severity.

Please note table has been reprinted with permission from publisher and van Bezooijen et al. (2015).

**Table 2:** Half-life of Traditional Antipsoriatic Drugs

Drug	Half-life
Methotrexate	3-10 hours (lower dose) 8-15 hours (higher dose)
Azathioprine	26-80 minutes (azathioprine) 3-5 hours (drug plus metabolites)
Acitretin	49 hours
Cyclosporine	Variable (about 24 hours)
Hydroxyurea	3-4 hours

**Table 3:** Half-life of Biologics Used for PsO

Drug	Half-life
Infliximab	8-10 days
Adalimumab	10-20 days
Etanercept	3-5.5 days
Ustekinumab	15-32 days (average 3 weeks)
⁎Efalizumab	5 days

⁎ No longer on the market, voluntarily pulled by Genentech due to increased risk of JC polyomavirus (1 in 500).

**Table 4:** Route of administration and dosing schedule for biologics

Drug	Route of administration	Dosing schedule
Infliximab	Intravenous	5 mg/kg given as an IV induction regimen at 0, 2, and 6 weeks followed by a maintenance regimen of 5 mg/kg every 8 weeks thereafter
Adalimumab	Subcutaneously	Initial dose of 80 mg, followed by 40 mg given every other week starting one week after the initial dose.
Etanercept	Subcutaneously	Initial: 50 mg subcutaneously twice weekly (administered 3 to 4 days apart) for 3 months. Alternatively, starting doses of 25 mg to 50 mg per week have been shown to be effective. Maintenance: 50 mg subcutaneously once weekly.
Ustekinumab	Subcutaneously	100 kg or less: Initial dose: 45 mg subcutaneously once initially and 4 weeks later Maintenance dose: 45 mg subcutaneously once every 12 weeks Greater than 100 kg: Initial dose: 90 mg subcutaneously once initially and 4 weeks later Maintenance dose: 90 mg subcutaneously once every 12 weeks
Secukinumab	Subcutaneously	Recommended dosage is 300 mg by subcutaneous injection at weeks 0, 1, 2, 3, and 4 followed by 300 mg every 4 weeks. For some patients, a dose of 150 mg may be acceptable.
⁎Efalizumab	Subcutaneously	150 mg subcutaneously once a week

⁎ No longer on the market, voluntarily pulled by Genentech due to increased risk of JC polyomavirus (1 in 500).
